# Aerobic Bacterial Profile of Sepsis and Its Antibiotic Susceptibility Pattern Among Patients in a Rural Tertiary Care Center

**DOI:** 10.7759/cureus.49942

**Published:** 2023-12-05

**Authors:** Anitha Deva, Namitha B N

**Affiliations:** 1 Microbiology, Sri Devaraj Urs Academy of Higher Education and Research, Kolar, IND

**Keywords:** extended-spectrum beta-lactamase, multidrug resistance, gram negative bacteria, blood culture, blood stream infections

## Abstract

Background

Bloodstream infections (BSI) are one of the most life-threatening infections associated with high morbidity and mortality. Early diagnosis with appropriate and timely treatment improves the patient outcome. The recent surge in multidrug-resistant (MDR) strains is a matter of concern. This study aims to determine the bacterial etiology and antibiotic sensitivity pattern in BSI among different age groups.

Materials and methods

The microbiological data of blood culture and sensitivity between April 2019 and April 2021 were extracted from the laboratory records and analyzed for the bacterial profile and antibiotic sensitivity pattern.

Results

Out of the total 3893 blood cultures received during the study period from April 2019 to April 2021, 194 pathogens were isolated, accounting for a prevalence of 4.98%. Among 194 patients with culture-proven BSI, 54.12% (105/194) were adults, and 45.87% (89/194) were children. Of these 194 bacterial isolates, 58.76% (114/194) were gram-negative bacteria, and 41.24% (80/194) were gram-positive bacteria. With regard to the bacteria isolated, *Enterococcus* species with 23.71% (46/194) and *Acinetobacter* species with 22.16% (43/194) were the most common bacteria. The prevalence of MDR was 59.27% (115/194). Notable MDR types were methicillin-resistant *Staphylococcus aureus* (MRSA) in 15/22 (68.2%) and extended-spectrum beta-lactamase (ESBL) producers in 15/48 (31.25%) cases.

Conclusion

There is a significant geographical diversity of bacteria causing sepsis and their antibiotic susceptibility pattern. Recent trends show that multidrug-resistant gram-negative bacilli are the predominant isolates causing BSI. Increased antibiotic resistance is leading to treatment failure and poor clinical outcomes. Hence, there is a need to monitor antibiotic resistance among patients with BSI.

## Introduction

Bloodstream infections, also known as sepsis, are severe life-threatening infections with a mortality rate of up to 50% [1]. At any given point of time, millions of people worldwide are affected by sepsis. Timely diagnosis with prompt and appropriate treatment is essential to reduce morbidity and mortality [1]. There has been a varied trend among patients with BSI regarding the age group affected, type of microorganism, and antimicrobial susceptibility pattern based on the epidemiologic and geographic features. The geriatric and pediatric age groups are the more vulnerable populations with increased infection rates associated with increased mortality, length of hospital stays, and hospital costs [2].

Pathogens that cause infection in different age groups vary. Among neonates, group B* Streptococcus* (GBS) and *Escherichia coli* are the most common pathogens in early-onset sepsis, whereas *Klebsiella pneumoniae* is the most common pathogen in late-onset sepsis [3]. Among older children and adults, gram-negative bacteria are more common pathogens than gram-positive bacteria. Microbiological diagnosis of BSI is crucial for identifying pathogens and determining the antimicrobial susceptibility pattern for pathogen-directed antimicrobial therapy. Blood culture and sensitivity remain the gold standard microbiological test for accurately identifying pathogens [4]. The automated method is easy to use, cost-effective, and reliable and has further reduced the turnaround time for microbiological diagnosis. Though recent advances in molecular techniques in the microbial diagnosis of infections have increased sensitivity and specificity for other infectious diseases, they have limited applicability in BSI because of the effectiveness of automated blood culture and sensitivity methods [5].

Antimicrobial resistance among microorganisms causing infections has recently become a significant public health problem due to the inappropriate and irrational use of antibiotics, which holds good even for BSI [6]. The local data on the microbial profile of BSI and their antimicrobial susceptibility pattern helps to formulate institutional antibiotic policy. The local antibiotic policy can significantly increase the survival rate among patients with BSI by guiding effective empirical antimicrobial therapy and reducing the development of antimicrobial resistance among pathogens [7]. 

This study aimed to determine the aerobic bacterial isolates responsible for septicemia in different age groups and their antibiotic sensitivity pattern at a tertiary care hospital, which in turn could contribute to formulating antibiotic policy for BSI at our institution.

## Materials and methods

This retrospective study was conducted in the Department of Microbiology at R.L. Jalappa Hospital and Central Diagnostic Laboratory Services, the unit of Sri Devaraj Urs Academy of Higher Education and Research (SDUAHER), Kolar. The study was approved by the Institutional Ethics Committee (DMC/KLR/IEC/229/2021-22). Data on the number of blood samples received for culture and sensitivity at a microbiology laboratory between April 2019 to April 2021 were extracted from the laboratory records. 

Blood samples collected under aseptic conditions were inoculated immediately into the blood culture bottle (aerobic blood culture bottle). Once received in the microbiology laboratory, the blood culture bottles were loaded into the BACTALERT-480 Automated blood culture system (bioMérieux, Marcy-l'Étoile, France). The bacterial isolates were identified from the positive vial by gram stain, colony morphology on blood agar, chocolate agar, and MacConkey agar, and by standard biochemical tests [8].

Antibiotic susceptibility testing was done by the modified Kirby-Bauer disc diffusion method by using Muller-Hinton agar. The bacterial isolates were tested for the specific panel of antibiotics and interpreted as sensitive and resistant based on the Clinical & Laboratory Standards Institute (CLSI) guidelines [9]. For Vancomycin and Colistin, minimum inhibitory concentration methods were used for sensitivity testing as per CLSI guidelines [9]. Based on antibiotic sensitivity patterns, the isolates were categorized into multidrug-sensitive (Multi S), monodrug-resistant (MoDR), multidrug-resistant (MDR), and extensively drug-resistant (XDR) based on standard definitions. Multi S are susceptible to all antibiotic classes, MoDR are resistant to single antibiotic class, MDR are resistant to at least one agent in three or more antimicrobial categories, XDR are non-susceptible to at least one agent in all but two or fewer antimicrobial categories (i.e., bacterial isolates remain susceptible to only one or two categories)[10].

Statistical analysis

Data was analyzed using SPSS 22 version software (IBM Inc., Armonk, New York). Categorical data was represented in the form of Frequencies and proportions. Microsoft Excel and Word (Micrososft, Redmond, Washington) were used to obtain various types of graphs.

## Results

**Figure 1:**
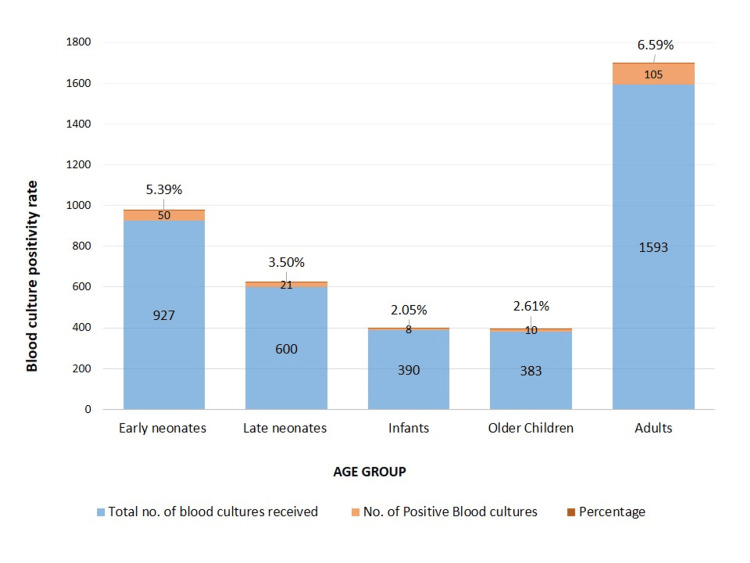
Distribution of Blood culture samples and their positivity rate among the different age groups

During the study period, 3893 blood samples were received for blood culture and sensitivity from the clinically diagnosed cases of primary BSI from the inpatients admitted to various wards like ICU, NICU, and others. Out of 3893 samples, 194 yielded the growth of pathogens, accounting for a prevalence of 4.98%. The distribution of blood culture samples and their positivity rate among different age groups is shown in Figure 1.

**Figure 2:**
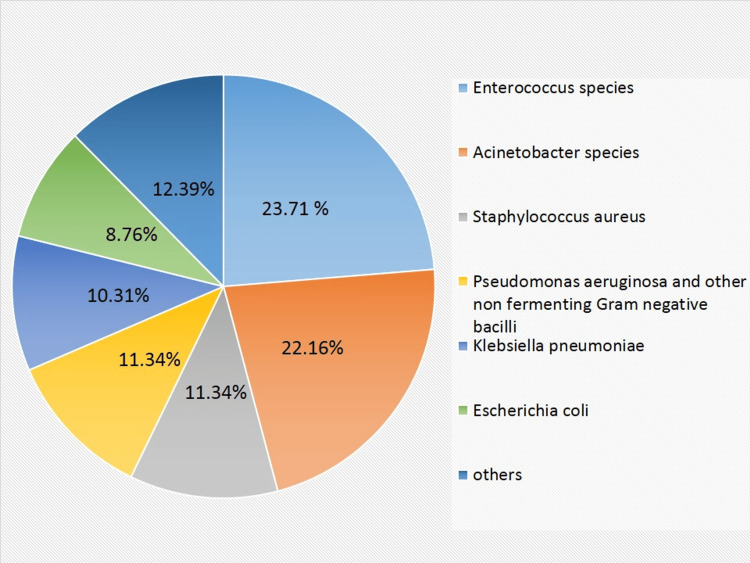
Frequency of bacterial isolates

Of the 3893 blood samples, 927, 600, 390, 383, and 1593 were from early neonates (up to seven days after birth), late neonates (eight to 28 days after birth), infants, older children, and adult patients, respectively. The blood culture positivity rate was 5.39% (50/927), 3.5% (21/600), 2.05% (8/390), 2.61% (10/383), and 6.59% (105/1593) among early neonates, late neonates, infants, older children, and adult patients, respectively. Of the 194 positive blood culture patients, 50 (25.77%) were early neonates, 21 (10.82%) were late neonates, 8 (4.12%) were infants, 10 (5.15%) were older children, and 105 (54.12%) were adults. The microbial profile of BSI is shown in Figure 2.

As a whole, 58.76% (114/194) of BSI were due to gram-negative bacteria, whereas 41.24% (80/194) were due to gram-positive bacteria. The most common bacteria isolated was *Enterococcus* species in 23.71% (46/194) of blood culture-positive patients, followed by *Acinetobacter species* in 22.16% (43/194), *Staphylococcus aureus* in 11.34% (22/194), *Klebsiella pneumoniae* in 10.31% (20/194) and *Escherichia coli* in 8.76% (17/194) of the patients. Nonfermenting gram-negative bacilli (NFGNB) and *Pseudomonas aeruginosa* were isolated from 6.19% (12/194) and 5.15% (10/194) of patients, respectively. The other less commonly isolated bacteria constituted 12.39% (24/194) of the isolates and included *Beta hemolytic Streptococcus, Enterobacter species, Streptococcus viridans, Serratia marcescens, Streptococcus pneumoniae, Klebsiella oxytoca, Salmonella Typhi, *and* Providencia species.*

The distribution of bacterial pathogens among different age groups is depicted in Table 1. 

**Table 1:** Distribution of blood culture isolates in different age groups

Organism	Total N (%)	Early-onset sepsis N (%)	Late-onset sepsis N (%)	Infants N (%)	Children N (%)	Adolescents N (%)	Adults N (%)
Gram-negative bacteria	114 (58.76)	31 (62)	13 (62)	6 (75)	7 (70)	0 (0)	57 (54.2)
*Acinetobacter *species	43 (22.16)	18 (36)	5 (23.8)	4 (50)	2 (20)	0 (0)	14 (13.3)
Escherichia coli	17 (8.76)	3 (6)	0 (0)	0 (0)	1 (10)	0 (0)	13 (12.3)
Klebsiella pneumoniae	20 (10.31)	5 (10)	3 (14.2)	0 (0)	1 (10)	0 (0)	11 (10.4)
Klebsiella oxytoca	2 (1.03)	0 (0)	0 (0)	0 (0)	0 (0)	0(0)	2 (1.9)
*Enterobacter *species	5 (2.58)	1 (2)	0 (0)	0 (0)	0 (0)	0 (0)	4 (3.8)
Serratia marcescens	3 (1.55)	1 (2)	1 (4.7)	0 (0)	1 (10)	0 (0)	0 (0)
*Providencia *species	1 (0.52)	0 (0)	0 (0)	0 (0)	0 (0)	0 (0)	1 (0.9)
Salmonella typhi	1 (0.52)	0 (0)	0 (0)	0 (0)	0 (0)	0 (0)	1 (0.9)
Pseudomonas aeruginosa	10 (5.15)	1 (2)	3 (14.2)	0 (0)	0 (0)	0 (0)	6 (5.7)
Gram-negative non-fermenters	12 (6.19)	2 (4)	1 (4.7)	2 (25)	2 (20)	0 (0)	5 (4.7)
Gram-positive bacteria	80 (41.24)	19 (38)	8 (38)	2 (25)	3 (30)	0 (0)	48 (45.7)
*Enterococcus *species	46 (23.71)	13 (26)	3 (14.2)	1 (12.5)	2 (20)	0 (0)	27 (25.7)
Staphylococcus aureus	22 (11.34)	1 (2)	3 (14.2)	1 (12.5)	1 (10)	0 (0)	16 (15.2)
Beta hemolytic *Streptococcus*	7 (3.61)	4 (8)	0 (0)	0 (0)	0 (0)	0 (0)	3 (2.8)
Streptococcus viridans	3 (1.55)	1 (2)	1 (4.7)	0 (0)	0 (0)	0 (0)	1 (0.9)
Streptococcus pneumoniae	2(1.03)	0 (0)	1 (4.7)	0 (0)	0 (0)	0 (0)	1 (0.9)
Total	194 (100)	50 (100)	21 (100)	8 (100)	10 (100)	0 (100)	105 (100)

*Acinetobacter *species was the predominant isolate among children, followed by *Enterococcus *species and *Klebsiella pneumoniae*; 36% of early-onset sepsis, 23.8% of late-onset sepsis, 50% of BSI in infants, and 20% in children aged more than one year were due to *Acinetobacter *species. *Enterococcus *species was isolated in 26% of cases of early-onset sepsis, 14.2% of cases in late-onset sepsis, 12.55% in infants, and 20% in older children. In adult patients with BSI, *Enterococcus* (25.7%) was the most common isolate, followed by *Staphylococcus aureus* (15.2%), *Acinetobacter species* (13.3%), *Escherichia coli* (12.3%) and *Klebsiella pneumoniae *(10.4%). 

**Figure 3:**
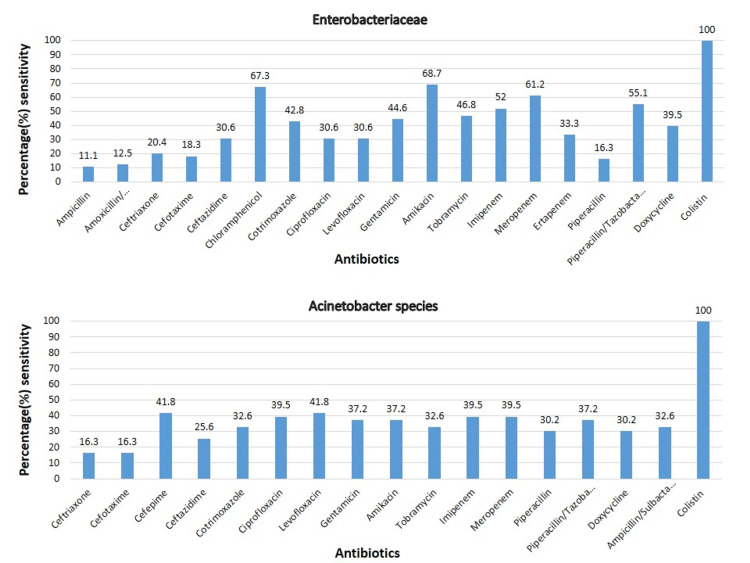
Antibiotic sensitivity pattern of Enterobacteriaceae and Acinetobacter species

**Figure 4:**
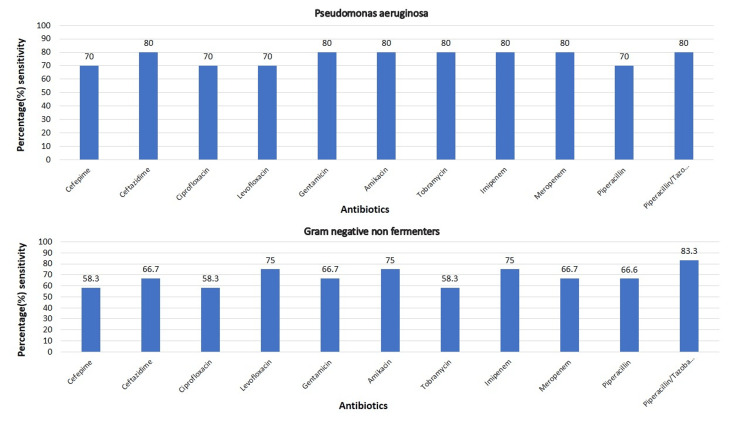
Antibiotic sensitivity pattern of Pseudomonas and gram-negative non-fermenters

**Figure 5:**
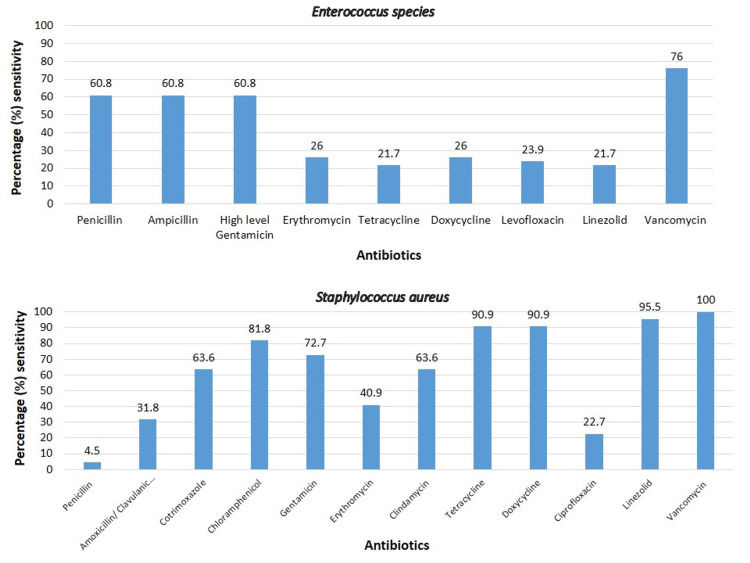
Antibiotic sensitivity pattern of gram-positive organisms

The antibiotic sensitivity pattern of gram-negative and gram-positive bacteria are shown in Figure 3, Figure 4, and Figure 5, respectively.

Enterobacteriaceae group of organisms showed susceptibility range from 11% to 100%. The lowest sensitivity was to ampicillin (11 %), and the highest was to colistin (100 %); next to colistin, >50% sensitivity was to amikacin, chloramphenicol, piperacillin/tazobactam, meropenem, and imipenem. Less than 50% sensitivity was found to tobramycin, gentamicin, cotrimoxazole, doxycycline, ertapenem, ceftazidime, ciprofloxacin, levofloxacin, ceftriaxone, cefotaxime piperacillin, amoxycillin/ clavulanic acid, and ampicillin.

The sensitivity of *Acinetobacter *species ranged from 16% to ceftriaxone and cefotaxime and 100% to colistin. The sensitivity to other antibiotics apart from colistin was <50%. Among the *Pseudomonas aeruginosa* and other NFGNB, the highest sensitivity was seen to piperacillin/tazobactam (81.81%), and the sensitivity to other antibiotics ranged from 50 to 80%.

Among the gram-positive bacteria, the sensitivity of *Enterococcus *species ranged from 76% to 21%; vancomycin sensitivity was 76%, followed by penicillin, ampicillin, and high-level gentamicin at 60.8%, erythromycin, and doxycycline at 26%, levofloxacin at 23.9% and linezolid at 21.7%. The sensitivity of* Staphylococcus aureus* ranged from 100% to vancomycin and 4.5% to penicillin. The sequence of sensitivity after vancomycin was to linezolid (95.5%), tetracycline and doxycycline at 90.9%, chloramphenicol (81.8%), gentamicin (72.7%), clindamycin and cotrimoxazole (63.6%), erythromycin (40.9), amoxicillin/ clavulanic acid (31.8%), ciprofloxacin (22.7%) and penicillin (4.5%). The sensitivity of *Streptococcus *species ranged from 100% to vancomycin, linezolid, penicillin, ampicillin, ceftriaxone, and cefotaxime, followed by 83.3% to cotrimoxazole, chloramphenicol, clindamycin, doxycycline and levofloxacin, 58.3% to gentamicin, erythromycin, tetracycline, and ciprofloxacin.

**Figure 6:**
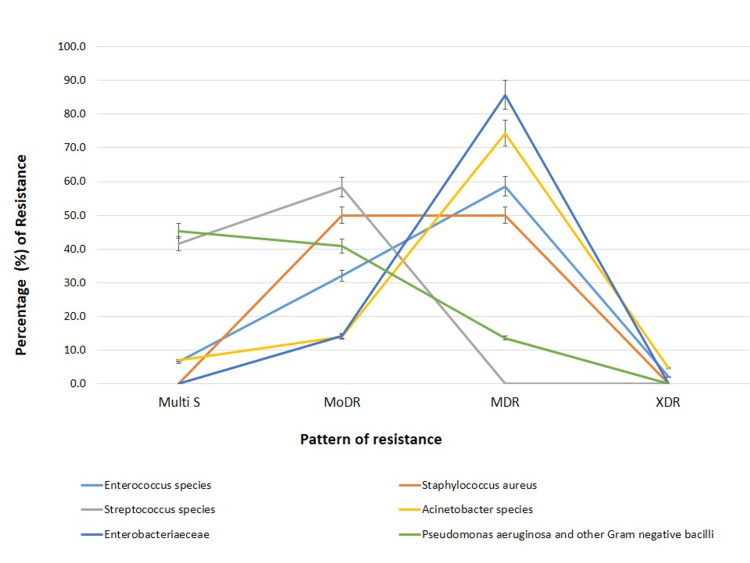
Frequency of Multi S, MoDR, MDR, and XDR among isolated bacteria Multi S - multidrug sensitivity, MoDR - monodrug resistance, MDR - multidrug resistance, XDR - extensive drug-resistance

The prevalence of different types of drug resistance patterns among the bacterial isolates is depicted in Figure 6.

XDR was seen in 5% of *Acinetobacter spp.* and 2% of *Enterococcus spp.* The prevalence of MDR was maximum among Enterobacteriaceae bacteria, accounting for 86%, followed by 74.4% among *Acinetobacter spp.*, 58.6% in *Enterococcus spp*., 50% in *Staphylococcus spp*., and 13.6% in *Pseudomonas aeruginosa* and other NFGNB. MoDR was present in 58.3%, 50%, 40.9%, 32%, 14%, and 13.9% of *Streptococcus, Staphylococcus aureus, Pseudomonas*, and NFGNB, *Enterococcus*, Enterobacteriaceae and *Acinetobacter spp.,* respectively. With regard to MultiS organisms, none of the *Staphylococcus* and Enterobacteriaceae were Multi S types. Multib S prevalence of 45.4% was found in *Pseudomonas* followed by 41.6% among *Streptococcus spp.* Only 6.9% of *Acinetobacter spp.* and 6.5% of *Enterococcus *were Multi S types.

## Discussion

Bloodstream infections are one of the most life-threatening infections in man. The pathogenic spectrum and the antibiotic susceptibility pattern are crucial in formulating local antibiotic policy that guides the effective management of these infections by appropriate empirical antibiotic therapy. The overall prevalence rate of BSI was 4.98% in our center, which is much lower compared to other studies, with recorded prevalence rates varying from 6.8% to 59% [11-13]. This could be because of the high probability of prior antibiotic use among patients elsewhere before getting admitted to our tertiary care center, which might have decreased the chance of isolation of bacteria from the blood samples of these patients. 

Among the patients with microbiologically confirmed BSI in our hospital, 54.12% were adult patients, and 45.86% were children, including infants. In a study done in Turkey by Akgun et al., 85.5% of the patients were adults, whereas 14.5% were pediatric patients [5]. In contrast, in a study among the population of the Republic of Kazakhstan, it was found that 34.8% of the patients with BSI were adults and 65.1% were children [14]. We found that the blood culture positivity rate was less in the pediatric age group than in adults. However, in a teaching hospital in Ghana, the highest positivity rate was found in infants (20.9%), followed by the elderly (13.3%), children (8.9%), and adults (7.2%) [15]. Similarly, in a study from India, 12.9% of blood culture positivity was found in Infants, 55% in elderly patients, 6.9% in children, and 6.72% in adults [11]. In the present study, the reason for the low positivity rate in neonates, infants, and children could be due to inappropriate sample collection procedures owing to the difficulty in collecting blood samples for blood culture at our institution because of increased workload and patient factors. An effective sample collection procedure for blood culture significantly influences the likelihood of pathogen isolation [15].

Gram-negative bacteria were the most predominant isolates in our study compared to gram-positive bacteria, accounting for 58.76% of BSI. This is in concordance with findings from other studies, which showed 62.2%, 65.8%, and 59.8 % of BSI due to gram-negative bacteria, and 36.4%, 38.8 %, and 34.2% due to gram-positive bacteria [4, 11, 14]. In contrast, gram-positive bacteria predominated at the rate of 60% in a study done by Thakur et al. [16]. This difference can be attributed to various factors like geographical distribution, ethnicity, and endemicity [4]. Though gram-negative bacteria were the predominant isolates as a whole, individually, *Enterococcus *was the most common isolate in 23.71% of the cases. The second most common isolate was the *Acinetobacter species*. Khurana et al. reported coagulase-negative* Staphylococci *(20.3%) and *Acinetobacter *species as the most common blood culture isolates [17]. A study by Kolesnichenko et al. revealed *Staphylococcus epidermidis *(35.5%) and *Staphylococcus aureus *(21.7%) as the most prevalent isolates in children and adult populations, respectively [14]. *Staphylococcus aureus *(45.6%) was the most common among pediatric patients, while *Salmonella enterica* (28.3%) was the most common among adult patients in a study by Parajuli et al. [11]. In our study, *Acinetobacter *species were the predominant isolate in neonates and infants, but *Enterococcus species* were more prevalent in the adult group. Reported risk factors for BSI due to* Enterococcus *species include advanced age and comorbid conditions [18].

Similar to our study, *Acinetobacter *species was the most common isolate in neonates in studies done by Ansari et al. [19]. Contrary to this, *Acinetobacter *species were the least prevalent bacteria among neonates at a rate of 4% in a study done in South Africa [20]. *Acinetobacter* are present ubiquitously and are the most enduring in the environment. Reported risk factors for septicemia due to *Acinetobacter* are inadequate infection control practices, prior antibiotic usage, prolonged hospitalization, high colonization pressure, and enteral feeding [19]. Antibiotic resistance among these isolates of BSI is one of the greatest threats, increasing the mortality rate due to the lack of available effective antibiotics according to the patient's need.

MDR strains were most prevalent in our study among the *Enterobacteriaceae *family (86%), *Acinetobacter *species (74.40%), *Enterococcus *species (58.60%), and *Staphylococcus aureus* (50%), similar to another study [21]. In our study, *Acinetobacter *showed 100% sensitivity to colistin and <50% sensitivity to other antibiotics, similar to the survey by Mahich et al., where 75% of them were MDR strains [22].

The WHO's critical priority pathogen list for India includes carbapenem-resistant *Acinetobacter *species [23]. Carbapenem resistance among *Acinetobacter *species has emerged over the years, as reported in various other studies [24]. The fact that 5% of the XDR *Acinetobacter* strains are solely sensitive to colistin in our study signifies how serious the drug resistance problem is and mandates constant vigilance. *Acinetobacter* is known for its rapid genetic changes and acquiring foreign genetic material through plasmids, resulting in its evolution and survival, which is the critical factor responsible for becoming resistant to antibiotics [25].

In our study, 31.25% were ESBL producers, and next to colistin, amikacin showed the highest sensitivity, similar to the study done in the Aljouf region of Saudi Arabia, where 23.3% were ESBL producers [4]. A study by Devanga Ragupathi et al. showed a similar pattern of antibiotic sensitivity of the Enterobacteriaceae family when the study included *Escherichia coli *alone, and 64% of them were ESBL producers with Amikacin showing the highest sensitivity [26]. Similarly, in China, Amikacin resistance remained low during the eight-year study, while there was an increase in the trends of resistance among the Cephalosporins and Beta-lactam with Beta-lactamase inhibitor combinations [24].

Other studies also report ampicillin, ceftriaxone, and cefotaxime being the most resistant antibiotics in the *Enterobacteriaceae *family, comparable to our study [17, 24]. MDR strains were highest among the *Enterobacteriaceae *family at 86% in our study. Multidrug-resistant *Escherichia coli* and *Klebsiella pneumoniae* were 78.3% and 49.2%, respectively, by Bandy et al. [4]. The increased usage of third-generation cephalosporins as the empiric medication in suspected sepsis patients, which has resulted in selection pressure leading to resistance, may cause this pattern of antibiotic resistance among the *Enterobacteriaceae *family of bacteria. In these situations, carbapenems are a preferable alternative. In contrast to prior research where carbapenem resistance grew over time among *Pseudomonas aeruginosa*, our investigation found that *Pseudomonas aeruginosa* and other gram-negative non-fermenters were the most sensitive strains, with 40.40% of the strains showing Multi S patterns [24]. Among gram-positive bacteria, *Streptococcus *species were the most sensitive strains.

Even though gram-positive bacteria are less common than gram-negative bacteria, antibiotic resistance among *Enterococcus* and *Staphylococcus *species is considerable. In our study, 68.2% of the *Staphylococcus aureus* were MRSA, and the most sensitive antibiotics against them were vancomycin, linezolid, tetracyclines, and chloramphenicol. *Staphylococcus aureus* susceptibility patterns across blood isolates from different studies were comparable, according to the Antimicrobial Resistance Surveillance and Research Network (AMRSN) annual report of 2020, with 54.4% of MRSA [27]. In our study, 26% of *Enterococcus* had vancomycin resistance. Other studies show VRE between 10% and 29% [28, 29]. In our investigation, 78.2% of the *Enterococcus* strains were resistant to linezolid. In contrast, a study by Jabbari Shiadeh et al. found that *Enterococcus *species had the lowest levels of linezolid resistance [30].

For the BSI, in the present scenario with increasing resistance to β-lactam antibiotics and other first-line drugs like fluoroquinolones and tetracyclines, carbapenems combined with aminoglycosides are recommended as the first-line antibiotics. However, because of emerging resistance to carbapenems, the last resort drug is colistin. A similar finding was noted in our study as well. This recommends an effective antimicrobial stewardship program in healthcare settings.

Limitations of the study

Since this is a retrospective study, clinical data and outcomes could not be captured. In addition, the time to positivity rate and blood culture contamination rate could not be analyzed and estimated.

## Conclusions

The most common isolates in primary BSI included *Acinetobacter *species, *Enterococcus *species, *Klebsiella pneumoniae*, *Escherichia coli*, and *Staphylococcus aureus*. The finding of the shift from MoDR to XDR among the bacterial isolates, more so with gram-negative bacilli than gram-positive cocci in our center, calls for appropriate empirical management of patients with BSI. The emergence of MDR and XDR strains, particularly in the more vulnerable populations, such as neonates, infants, and the elderly, is of high concern as it makes treatment a significant challenge. This calls for close monitoring and targeted infection prevention control practices to stop the spread of these infections and a rigorous antimicrobial stewardship approach due to the lack of newer and effective antibiotics.
